# Development of the Intersectoral Care Reported by Patients Survey for Primary and Oral Healthcare

**DOI:** 10.5334/ijic.8933

**Published:** 2025-07-23

**Authors:** Kirsten P. J. Smits, Marieke M. van der Zande, Reanne Hek, Pim W. J. J. Assendelft, Fred R. Rozema, Kenneth R. Wright, Maria C. Dolce, Christine A. Riedy, Jane R. Barrow, Fabian Hüttig, Stefan Listl

**Affiliations:** 1 Department of Quality and Safety of Oral Health Care, Radboud University Medical Center, Nijmegen, The Netherlands; 2 Department of Public Health, Policy & Systems, Institute of Population Health, University of Liverpool, United Kingdom; 3 Department of Primary and Community Care, Radboud University Medical Centre, Nijmegen, The Netherlands; 4 Department of Oral Medicine, Academic Center for Dentistry, Amsterdam, The Netherlands; 5 Department of Oral and Maxillofacial Surgery, Amsterdam University of Amsterdam, Amsterdam, The Netherlands; 6 Dental Care Services, Kaiser Foundation Health Plan of the Northwest, Portland, OR, United States; 7 Department of Comprehensive Care, Tufts University School of Dental Medicine, Boston, MA, United States; 8 Oral Health Policy and Epidemiology, Harvard School of Dental Medicine, Boston, MA, United States; 9 Department of Prosthodontics, Centre of Dentistry, Oral Medicine, and Maxillofacial Surgery, University Hospital Tübingen, Tubingen, Germany; 10 Section for Oral Health, Heidelberg Institute of Global Health, Heidelberg University Hospital, Heidelberg University, Heidelberg, Germany

**Keywords:** intersectoral care, dental care, primary health care, patient perceptions, quality improvement, doctor-patient communication

## Abstract

**Introduction:**

Oral and general health are closely related, and many oral and chronic-systemic diseases have the same risk factors. However, in most countries dental and general health care systems are separated. Integration of care for patients with closely related, multiple conditions is therefore important to ensure they receive the best quality of care. We aimed to develop a short and easy-to-understand survey to be used by patients to assess integration of dental and primary health care through their perceptions.

**Methods:**

This study used both qualitative and quantitative methods. A modified online Delphi method was used for development of the survey. A panel consisting of seven experts assessed the survey in three rounds. The experts were based in the United States, Germany and the Netherlands and had different professional backgrounds, including general practice and dentistry. The initial framework and set of proposed questions were based on a previous survey with a similar focus. During two discussion rounds and one online round the framework and the questions were assessed and modified by the experts. The survey was then assessed for understandability among 18 patients and thereafter piloted among 199 patients.

**Results:**

The final questionnaire derived from the consensus procedure contains thirteen questions which address the following five domains: patients’ wishes, expectations, awareness and concerns regarding communication between health care providers; patients’ perception of health care providers’ knowledge; health care provider-patient communication about health status; utilization of health care; and self-rated health. The consensus procedure also yielded improvements in the understandability of survey items: one survey item was changed from a multiple-choice question into a yes/no question, answer options were added to three other survey items, four survey items were slightly changed in wording, and five items remained unchanged.

**Conclusion:**

We developed the short, easy-to-understand Integrated Care Reported by Patients Survey to assess integration of dental and primary health care as perceived by patients. In the future, the developed survey is intended to be tested for validity and translated to other languages. The survey provides opportunities for usage in research and as a tool in quality improvement and feedback systems for health care providers.

## Introduction

The prevalence of oral diseases is high with billions of people suffering from oral conditions [1]. Globally, the total disability-adjusted life years due to dental conditions have been increasing since 1990. The economic burden of oral diseases is large, both on the individual and the societal level [2]. In addition to the direct economic burden, oral diseases also have a significant impact on a patient’s quality of life [3,4,5].

Oral health has been shown to be linked with general health, and vice versa. More and more evidence is found for associations between oral diseases and chronic-systemic diseases, such as diabetes, cardiovascular diseases and respiratory diseases [6,7,8]. Diabetes has a direct relationship with periodontitis, with increased risk of periodontitis in diabetic patients and periodontitis negatively affecting glycemic control. It is thought that the inflammatory nature of both conditions plays a role in this relation [9]. Inflammatory pathways also play a role in the relation between periodontitis and atherosclerosis, which in turn leads to cardiovascular diseases [10]. Besides biological pathways, associations may also be explained indirectly by common risk factors. Many chronic-systemic diseases and oral diseases have the same risk factors, such as excess sugar consumption and smoking. Reducing these risk factors could improve both oral and general health [11]. In addition, prescribed and over-the-counter medication can lead to adverse oral effects such as discoloration, caries and erosion [12]. In conclusion, there are multiple reasons to promote integrated dental and general health care.

A recent study showed that primary care physicians can play a role in oral health care by assessing the risk of oral disease, performing clinical evaluations of the mouth, providing preventive interventions such as fluoride varnish, and providing targeted education to their patients about oral health and dental hygiene [13]. Similarly, it has been proposed that oral health care providers can play a role in screening for chronic-systemic diseases, as well as in prevention through promoting healthy behaviors [14]. Increased integration between primary medical care and oral health care can therefore improve prevention, diagnosis, treatment, and monitoring of patients with both oral and chronic-systemic diseases [14,15]. However, the separation of the medical and dental curriculum keeps contributing to a culture of disintegration between the dental and medical professions with limited expectations for collaborating with one another [16].

A key element in integrated health care is the central role of the patient [17]. According to Suter et al. one of the key elements of successful health system integration is “patient focus”, or that the patient should be at the center of the integrated care [18]. Positive patient perspectives can be used as indicators for successful integrated care or successful interventions to promote integrated care. Studies have shown that patient-centeredness is related to better clinical outcomes and reduced care utilization as well as to better understanding of diseases and improved self-management of diseases among patients [19,20]. Several questionnaires have been developed to assess the integration of care [21,22,23,24]. However, some of these focus on the professional perspective instead of the patient’s perception [24]. Other questionnaires focus on identifying which kind of integrated care occurred [22,23]. Only the Patient Perceptions of Integrated Care (PPIC) survey was developed to assess the integration of care as perceived by patients, focusing on integration between primary and secondary care [21]. Better performance on this survey was associated with lower health care utilization [25]. However, the PPIC survey includes eighty items, making it a long and time-consuming survey for patients to complete. Given that factors other than the patients’ perceptions such as governance structure and financial management are also important for promoting integrated care [18], a more concise patient survey instrument would be supportive of integrating various stakeholder perspectives on integrated care. Understanding the views of multiple stakeholders is particularly relevant in the context of dentistry and medicine which constitute distinct sectors within healthcare. In addition, a concise patient survey instrument on integrated care could also serve as a complement to instruments for measuring quality of life.

To this end, we aimed to develop a survey to assess intersectoral care as perceived by patients, which we call the Intersectoral Care Reported by Patients (ICRP) Survey. The survey should be (I) short in words and number of questions, (II) easy to understand for patients and (III) possibly function as an additive survey, to be used in combination with other patient-reported measures. The survey should be usable as a research instrument as well as a tool to evaluate and provide feedback to health care providers on the perceived delivered intersectoral care for primary and oral healthcare.

## Methods

This study using both qualitative and quantitative methods was conducted in two phases. In phase 1, a panel of experts assessed the proposed framework and questions using a modified online Delphi method in multiple rounds [26]. In phase 2, the developed prototypic survey was assessed for understandability by a panel of patients and then pilot tested among Dutch participants in general medical and dental practices. Based on the findings of this pilot final modifications were made by the research team.

### Phase 1

#### Development of framework and survey questions

Based on the framework used for the PPIC survey [21], a conceptual framework was developed which was intended to be simple, easy to understand, and having a focus on intersectoral care communication that could be reported by patients. The conceptual framework was developed by two researchers (KPJS, MMZ) and discussed with a third researcher (SL). Based on this conceptual framework and the previously developed PPIC survey, a preliminary version of the ICRP Survey was developed by the research team. The framework and the initial version of the ICRP survey were assessed and further developed by an international expert panel. The framework underlying the generation of items was intended to be simple, easy to understand and focus on what patients could report on the integration between oral and general health care. Therefore, the framework shows only partial overlap with the PPIC framework. The dimensions focused on patient-centeredness and communication with the patient were similar between PPIC and ICRP, while dimensions for health care utilization and self-rated health were new in our ICRP framework. These dimensions allow the ICRP Survey outcome to be adjusted for care utilization and self-rated health.

#### Expert panel

The panel members were international experts in the field of primary care, oral care, and academic leaders in medical-dental integration and patient-reported outcome measures. In total, seven experts from the United States, Germany and the Netherlands participated in the expert panel (WJJA, FRR, KRW, MCD, CAR, JRB, FH). They received the first version of the framework and the survey electronically along with an explanation and instruction of the process before the first round. The first round consisted of an online group discussion chaired by a moderator (MMZ). The group discussion started with an explanation of the aim of the survey. Thereafter, firstly the domains were discussed. The experts were invited to change, add, or remove domains. After consensus on the domains, the questions were discussed and the experts were again invited to change, add, or remove questions. When experts were unable to attend the group discussion, they were consulted individually by the research team. The survey and the framework were adjusted by the research team based on this first round.

In the second round, all questions proposed in the first round were assessed by the expert panel via an online survey. Some questions were included in different formats, presenting alternative wordings based on the first round. The questions were first rated on necessity using the question “Do you think the question … is necessary in the ICRP survey?” and scored on a 9-point Likert scale, where 1 reflects ‘completely unnecessary’ and 9 ‘absolutely necessary’. The ICRP questions either had a yes/no-format or a multiple-choice format. For the multiple-choice questions, the experts were asked “Do you agree with the answer categories for question …?”. If the experts indicated they did not agree, they were asked to suggest other answer categories. Furthermore, when multiple formats were proposed for one question, the experts were asked to select the format they preferred. For each question and for the whole survey, experts had the option to provide comments and propose changes or new questions. Finally, the experts were asked to indicate their top ten most important questions to be included in the ICRP Survey.

After the second round, the scores as given by the expert panel were analyzed. The questions were ranked, based on the frequency of being in the top ten most important items, and on the median and mean scores of necessity given by the experts. Comments provided by the experts were summarized and discussed by the research team. During this discussion, some questions were changed, based on experts’ feedback and a final number of questions was established.

The third round consisted of an online group discussion to reach consensus on the questions to be included in the ICRP Survey. This discussion was again chaired by MMZ. The experts received a summary of the results of the online round before the group discussion. The ranking of the questions was discussed, as well as the number of questions to be included in the ICRP and possible changes that were proposed in the online round. Experts who were unable to attend the group discussion were consulted individually by the research team. The final version, which was established during the third round, was then sent electronically to the expert panel for final approval.

### Phase 2

#### Understandability

In phase 2, the developed ICRP instrument was developed further in the Dutch setting. Two researchers independently translated the English version into Dutch and discussed their discrepancies until they agreed on translation. Thereafter, through feedback from a patient panel, the understandability of the ICRP Survey was assessed. Patients in this panel were recruited through the personal network of one researcher (RH), and through contacts of some of the panelists. They received a unique link to the ICRP Survey and were asked to assess the understandability. The introduction of the survey and each question were separately rated by using the question “Is it completely clear for you what is meant by this question?” and scored on a 9-point Likert scale (where 1 represents ‘completely unclear’ and 9 represents ‘absolutely clear’). Additionally, after each question participants were asked whether there were any unclear words or phrases. Suggestions and remarks could be made after each question. At the end of the questionnaire, participants were asked if they had any other suggestes changes for the ICRP survey. After round 1, the scores and suggestions were analyzed. Questions with a median score ≥7 were deemed understandable. Any question with a median score <7 showed the need for adjustment. In addition to the scores, any suggestions from the patient panel to revise the ICRP survey werediscussed by the researchers until consensus was reached about incorporating the suggested changes. The adjusted set of questions was similarly assessed in a second round. This cycle continued until all questions were deemed understandable.

#### Pilot test

A pilot test of the ICRP instrument was performed with focus on feasibility. Patients (≥18 years of age) were recruited at one general medical practice, two dental practices, and the social networks of two researchers (KPJS, RH). During the period of November and December of 2019 participants were asked to complete the ICRP Survey; a paper version at the medical and dental practices and an online version for the participants from the social network.

#### Statistical analysis

Descriptive statistics were used to describe the results of the pilot test. Frequencies and percentages, as well as means and standard deviations were reported for the questions of the ICRP Survey. When multiple answers were given to ICRP questions with a Likert scale, these answers were excluded from the analyses and assumed to be missing.

#### Involvement of people with lived experience

People with lived experience have been involved as follows: a panel consisting of patients assessed the understandability of the ICRP survey. Subsequently, the ICRP survey was pilot tested among patients in primary medical care and oral health care settings.

#### Ethics approval

No ethics approval was required for this study according to the applicable medical ethics regulations in the Netherlands (WMO).

## Results

### Phase 1

#### Framework and initial survey

The dimension definitions were discussed to ensure that the most relevant and necessary aspects were included in the ICRP framework. The five domains of the conceptual framework for the ICRP Survey were: (1) patients’ wishes regarding communication between health care providers; (2) patients’ perception of the health care provider’s knowledge; (3) provider-patient communication about health status; (4) utilization of health care; and (5) self-rated health. The first three dimensions included questions related to the actual perceived collaboration between health care providers, while the dimensions of health care utilization and self-rated health relate to the patients themselves. Low utilization of health care or poor self-rated health may possibly influence the responses of the patients towards the other dimensions. Therefore, these dimensions were added to distinguish between different patient groups when deemed necessary. In total, 11 questions were developed (Table 1). Some domains contained multiple similar questions to assess the domains on both general and oral health, or primary and oral health care.

**Table 1:** Domains of initial framework, first set of proposed questions and the final ICRP survey questions per domain.


DOMAINS		QUESTIONS	
	
CONCEPTUAL FRAMEWORK	FINAL FRAMEWORK	FIRST SET OF PROPOSED QUESTIONS	ICRP SURVEY QUESTIONSα

Patient wishes regarding communication between health care providers	Patient wishes, expectations, awareness and concerns/limitations regarding communication between health care providers	Do you want your health care providers to exchange information about your health?	How aware are you of possible relationships between your oral and general health? In general, how frequently do you want your health care providers to coordinate your care based on both your general and oral health? Which type of health information do you want your health care providers to communicate about?β

Perception of providers’ intersectoral knowledge	Patient perception of health care provider’s knowledge	Do you think your dentist is up-to-date regarding your general health? Do you think your general practitioner is up-to-date regarding your dental/oral health?	In your view, how much knowledge should your dentist or dental health care provider have about general health care? In your view, how much knowledge should your general practitioner or primary health care provider have about oral health care? My dentist or oral health care provider is aware of my medical history My general practitioner or primary health care provider is aware of my dental history

Communication from health care provider to patient about health status	Health care provider-patient communication about health status	Did your dentist ever discuss your general health with you? During your most recent dental visit, did your dentist ask about (changes in) your general health? Did your general practitioner ever discuss your dental/oral health with you? During the past year, did your general practitioner ask about (changes in) your dental/oral health?	During your most recent dental visit, did your dentist or oral health care provider ask about one of the following aspects?γ During the past year, did your general practitioner or primary health care provider ask about one of the following aspects?δ

Health care utilization for both sectors	Utilization of health care	When did you last visit a dentist? When did you last visit a general practitioner?	When did you last visit a dentist or oral health care provider? When did you last visit your general practitioner or primary health care provider?

Self-rated health	Self-rated health	How would you rate your dental/oral health? How would you rate your general health?	How would you rate your oral health? How would you rate your general health?


^α^ For the answering options of this version see Appendix III. ^β^ Types of health information: Recent diagnosis; Results of medical tests; Medication; Infectious diseases; Medical history; Social history; Emotional state, for example sadness; Mental health; Other, namely…; I do not want my health care providers to communicate; I do not know. ^γ^ The following aspects: Your medical history; Your social history; Visit to the general practice; Visit to the hospital; Reasons for visiting a health care provider; Any medical test results; Changes in your medication; Changes in your general health; other, namely …; None; I do not know or do not remember. ^δ^ The following aspects: Your dental history; Visits to the dentist or dental clinic; Reasons for visiting a dental health care provider; Dental screening/exam; Dental treatment; Problems with your mouth, teeth, gums or jaw; Other, namely…; None; I do not know or do not remember.

#### Expert panel

##### Round 1

Five out of the seven experts were able to attend the first online round of discussion. Changes were proposed to the conceptual framework, which are shown in Table 1. Fourteen new questions were added and for thirteen questions several formats were proposed. After the discussions the proposed survey consisted of 25 questions, including 44 formats (Appendix I).

##### Round 2

When asked their top ten most important questions, the experts chose one question five times. One question was chosen four times, five questions three times, nine questions two times and eighteen questions one time in the top ten of most important questions. The median scores for all questions were relatively high, with only five out of 44 questions with a median score below seven (Appendix II).

##### Round 3

Four experts attended round three in which the ranking of the questions was discussed. The expert panel decided that for each selected question about either the primary care or oral health care provider, the accompanying question for the other type of health care provider should also be selected in the ICRP Survey regardless of its ranking. The accompanying question was always the same question, but focused on the other health care provider. The highest ranked questions were focused on awareness and perceived necessity of integration of care, as well as specific questions about knowledge and awareness of the primary and oral health care provider (Table 1). The final ICRP Survey consisted of 13 questions (see Table 1) and was approved by all experts.

### Phase 2

#### Understandability

The introduction and questions of the ICRP Survey were successfully translated from English into Dutch. During the first round of the patient panel regarding understandability, 18 persons completed the survey. Of these 18, 13 participants completed the second round. In the first round, all questions had a median score ≥7 (Table 2). However, several suggestions were made for changes to the questions to improve understandability. The question regarding coordination of care (question 2) was changed from a multiple-choice question to a yes/no question. The neutral answer options of two similar questions regarding awareness of medical or dental history by the dental or primary health care provider (question 6 and 11) were supplemented with the words ‘I do not know’ (answer 3 on a 5-point scale from completely disagree to completely agree). Finally, the answer option ‘social history’ was added to the question regarding information requested by the dental health care provider (question 7). Four additional questions were slightly changed in wording (Table 2). The other questions did not receive additional comments and remained unchanged. After the second round, all questions had a median score of 9 and were deemed understandable (see Appendix III).

**Table 2:** Results of the two rounds with patient panel and adjustments made to the questions.


ORIGINAL ICRP SURVEY ITEMS	ROUND 1^§^	ROUND 2^§^	CHANGES (AS SUGGESTED BY PATIENT PANEL)	FINAL ICRP SURVEY QUESTION^α^

Introduction	8.5	9	- Addition of definition of integration of health care - Addition of definition of health care providers - Addition of definition of social history	–

1. Awareness of relations between oral and general health	8.5	9	- Addition of example	How aware are you of possible relationships between your oral and general health? (e.g. the influence of diabetes on gum health)

2. Frequency of coordinated care	8	9	- Yes/no-question instead of multiple-choice question - Addition of example	Do you want your health care providers to coordinate your care based on both your general oral health? (e.g. exchange all information they deem necessary)

3. Type of health information communicated	9	9	No changes	Which type of health information do you want your health care providers to communicate about?^β^

4. Knowledge dental health care provider about general health care	9	9	- Change general health care into general health - Addition of example	In your view, how much knowledge should your dentist or oral health care provider have about general health? (e.g. be aware of symptoms and diseases in the whole body, not the mouth)

5. Last visit dental health care provider	9	9	No changes	When did you last visit a dentist or oral health care provider?

6. Awareness dental health care provider of medical history	9	9	- Addition of example - Addition of ‘I do not know’ in answer options	My dentist or oral health care provider is aware of my medical history. (e.g. he/she knows which disease(s) I have and takes these into account)

7. Information requested by dental health care provider	9	9	- Answer option added	During your most recent dental visit, did your dentist or oral health care provider ask about one of the following aspects?^γ^

8. Self-perceived general health	9	9	No changes	How would you rate your general health?

9. Knowledge general practitioner about oral health	9	9	- Change oral health care into oral health - Addition of example	In your view, how much knowledge should your general practitioner have about oral health? (e.g. be aware of symptoms and diseases in the mouth)

10. Last visit general practitioner	9	9	No changes	When did you last visit your general practitioner or primary health care provider?

11. Awareness general practitioner of dental history	9	9	- Addition of example - Addition of ‘I do not know’ in answer options	My general practitioner or primary health care provider is aware of my dental history. (e.g. he/she knows problems I have in my mouth and takes these into account)

12. Information requested by general practitioner	9	9	No changes	During your most recent medical visit, did your general practitioner or primary health care provider ask about one of the following aspects?^δ^

13. Self-perceived oral health	9	9	No changes	How would you rate your oral health?


^α^ For the answering options of this version see Appendix III. ^β^ Types of health information: Recent diagnosis; Results of medical tests; Medication; Infectious diseases; Medical history; Social history; Emotional state, for example sadness; Mental health; Other, namely…; I do not want my health care providers to communicate; I do not know. ^γ^ The following aspects: Your medical history; Your social history; Visit to the general practice; Visit to the hospital; Reasons for visiting a health care provider; Any medical test results; Changes in your medication; Changes in your general health; other, namely …; None; I do not know or do not remember. ^δ^ The following aspects: Your dental history; Visits to the dentist or dental clinic; Reasons for visiting a dental health care provider; Dental screening/exam; Dental treatment; Problems with your mouth, teeth, gums or jaw; Other, namely…; None; I do not know or do not remember. ^§^ In Round 1, the rating was on necessity using the question “Do you think the question … is necessary in the ICRP survey?” and scored on a 9-point Likert scale, where 1 reflects ‘completely unnecessary’ and 9 ‘absolutely necessary’. In Round 2, the rating was on understandability using the question “Is it completely clear for you what is meant by this question?” and scored on a 9-point Likert scale (where 1 represents ‘completely unclear’ and 9 represents ‘absolutely clear’).

#### Pilot Study

In total, 199 participants filled in the ICRP Survey, of which 187 surveys were complete. Approximately two-thirds of the participants were females (n = 127, 64%), close to one-third were younger than 25 (n = 62, 31%), and the majority completed higher education, (n = 120, 60%). Results of ICRP survey pilot study can be found in Table 3. Most participants indicated that they would like their health care to be coordinated based on both their general and oral health (question 2, n = 161, 81%), only 13 (7%) did not want any type of health information communicated between health care providers (question 3). Most participants visited the dental or primary health care provider in the last 6 months (question 5 and 10 respectively, n = 139 (70%) and n = 114 (57%)). Most questions had good variability in the answers. According to participants, their primary health care provider rarely asked about their dental aspects (question 12, no aspects asked: n = 167 (84%), while the dental health care providers asked about medical aspects in just over half of the cases (question 7, no aspects asked: n = 89 (45%). There was variability in the assumed knowledge and awareness of the primary and dental health care provider (Table 3). The twelve incomplete questionnaires were all paper-based, and most of them were missing responses to the last questions, suggesting that they did not have time to complete the questionnaire. Four questionnaires included multiple answers to questions with Likert scales and were therefore excluded from the analyses and reported as missing in Table 3.

**Table 3:** Results of the ICRP survey in the pilot study (n = 199).


VARIABLES	N (%)

Age	

Younger than 25 years	62 (31.2)

Between 25 and 35	21 (10.6)

Between 36 and 45	18 (9.1)

Between 46 and 55	31 (15.6)

Between 56 and 65	39 (19.6)

Older than 65 years	23 (11.6)

Missing	5 (2.5)

Gender	

Female	127 (63.8)

Male	61 (30.7)

Other/prefer not to say	5 (2.5)

Missing	6 (3.0)

Income	

≤€20,000 gross per year	70 (35.2)

Between €20,000 and €50,000 gross per year	51 (25.6)

Between €50,000 and €100,000 gross per year	27 (13.6)

≥€100,00 gross per year	11 (5.5)

Prefer not to say/I do not know	32 (16.1)

Missing	8 (4.0)

Highest completed degree of education	

Primary education	3 (1.5)

Secondary education degree up to 4 years	22 (11.1)

Secondary education degree up to 6 years	46 (23.1)

Undergraduate degree or higher	120 (60.3)

Missing	8 (4.0)

ICRP survey	

1. How aware are you of possible relationships between your oral and general health? (e.g. the influence of diabetes on gum health)	

Not at all aware	14 (7.0)

Slightly aware	44 (22.1)

Somewhat aware	38 (19.1)

Moderately aware	55 (27.6)

Extremely aware	48 (24.1)

Missing	0 (0.0)

2. Do you want your health care providers to coordinate your care based on both your general and oral health? (e.g. exchange all information they deem necessary)	

Yes	161 (80.9)

No	20 (10.1)

I do not know	17 (8.5)

Missing	1 (0.5)

3. Which type of health information do you want your health care providers to communicate about?	

Recent diagnosis	130 (65.3)

Results of medical tests	100 (50.3)

Medication	146 (73.4)

Infectious diseases	112 (56.3)

Medical history	109 (54.8)

Social history	27 (13.6)

Emotional state	25 (12.6)

Mental health	37 (18.6)

Other	8 (4.0)

I do not want my health care providers to communicate	13 (6.5)

I do not know	7 (3.5)

Missing	0 (0.0)

4. In your view, how much knowledge should your dentist or oral health care provider have about general health? (e.g. be aware of symptoms and disease in the whole body, not the mouth only)	

Not knowledgeable at all	1 (0.5)

Slightly knowledgeable	42 (21.1)

Moderately knowledgeable	63 (31.7)

Very knowledgeable	67 (33.7)

Extremely knowledgeable	23 (11.6)

Missing	3 (1.5)

5. When did you last visit a dentist or oral health care provider?	

Less than 6 months ago	139 (69.9)

6–12 months ago	43 (21.6)

1–2 years ago	10 (5.0)

2–10 years ago	5 (2.5)

Never visited a dentist or dental health care provider in the last 10 years	2 (1.0)

Missing	0 (0.0)

6. My dentist or oral health care provider is aware of my medical history. (e.g. he/she knows which disease(s) I have and takes these into account)	

Strongly disagree	20 (10.1)

Disagree	39 (19.6)

Neither agree nor disagree (I do not know)	63 (31.7)

Agree	55 (27.6)

Strongly agree	21 (10.6)

Missing	1 (0.5)

7. During your most recent dental visit, did your dentist or oral health care provider ask about one of the following aspects?	

Your medical history	36 (18.1)

Your social history	8 (4.0)

Visit to the general practice	12 (6.0)

Visit to the hospital	13 (6.5)

Reasons for visiting a health care provider	10 (5.0)

Any medical tests results	8 (4.0)

Changes in your medication	36 (18.1)

Changes in your general health	69 (34.7)

Other	4 (2.0)

None	89 (44.7)

I do not know/I do not remember	11 (5.5)

Missing	3 (1.5)

8. How would you rate your general health?	

Poor	4 (2.0)

Fair	31 (15.6)

Good	89 (44.7)

Very good	49 (24.6)

Excellent	21 (10.6)

Missing	5 (2.5)

9. In your view, how much knowledge should your general practitioner have about oral health? (e.g. be aware of symptoms and diseases in the mouth)	

Not knowledgeable at all	3 (1.5)

Slightly knowledgeable	35 (17.6)

Moderately knowledgeable	62 (31.2)

Very knowledgeable	66 (33.2)

Extremely knowledgeable	28 (14.1)

Missing	5 (2.5)

10. When did you last visit your general practitioner or primary health care provider?	

Less than 6 months ago	114 (57.3)

6–12 months ago	45 (22.6)

1–2 years ago	18 (9.1)

2–10 years ago	16 (8.0)

Never visited a general practitioner or primary health care provider in the last 10 years	3 (1.5)

Missing	3 (1.5)

11. My general practitioner or primary health care provider is aware of my dental history. (e.g. he/she knows problems I have in my mouth and takes these into account)	

Strongly disagree	47 (23.6)

Disagree	49 (24.6)

Neither agree nor disagree (I do not know)	63 (31.7)

Agree	29 (14.6)

Strongly agree	7 (3.5)

Missing	4 (2.0)

12. During your most recent medical visit, did your general practitioner or primary health care provider ask about one of the following aspects? (See appendix III for the aspects)	

Your dental history	4 (2.0)

Visits to the dentist or dental clinic	3 (1.5)

Reasons for visiting a dental health care provider	4 (2.0)

Dental screening/exam	4 (2.0)

Dental treatment	4 (2.0)

Problems with your mouth, teeth, gum or jaw	9 (4.5)

Other	4 (2.0)

None	167 (83.9)

I do not know/I do not remember	12 (6.0)

Missing	3 (1.5)

13. How would you rate your oral health?	

Poor	3 (1.5)

Fair	45 (22.6)

Good	87 (43.7)

Very good	36 (18.1)

Excellent	25 (12.6)

Missing	3 (1.5)


## Discussion

This paper described the development of the Intersectoral Care Reported by Patients Survey (ICRP Survey) to assess patient perceptions on the integration of oral and primary health care. The framework for this survey consisted of five domains focusing on patient wishes, expectations, awareness and concerns or limitations regarding communication between health care providers; perception of health care providers’ knowledge; health care provider-patient communication about health status; utilization of health care; and self-rated health. After assessing the understandability amongst patients, the ICRP Survey in its final version consisted of a total of 13 questions.

### Practical implications of the ICRP Survey

The ICRP Survey may be used in feedback systems both to gain awareness for the importance of collaboration between general practitioners and dentists and to improve this collaboration. Previously, surveys have been developed to assess the integration of care, such as the Patient Perceptions of Integrated Care (PPIC) survey [21], the Provider and Staff Perceptions of Integrated Care (PSPIC) survey [24], and the Medical Home Care Coordination (MHCC) survey [22]. These and other surveys are focused on perceptions of health care staff rather than patients [24], on the actual occurrence of integrated care [23], or were developed for specific settings such as primary care practices involved in the Medical Home transformation [22]. The ICRP Survey is short in number of questions, containing only one-sixth of the number of questions in the PPIC survey, for example. Furthermore, the ICRP Survey is focused on how patients perceive integrated care, as well as to what extent patients prefer to receive integrated care. The ICRP Survey therefore contributes to the existing measures by having a patient-centered focus and being easy to use in addition to other measures.

One of the main aims was to make the ICRP Survey suitable for use in addition to other patient surveys. For example, it could be used alongside health-related quality-of-life surveys, such as the Short Form 36 Health Survey (SF-36), since the SF-36 is a more extensive measure of perceived health than the one question in the ICRP Survey regarding self-perceived health [27]. Combining the SF-36 with the ICRP Survey may provide researchers with the ability to distinguish between different health statuses or different components of health and compare this against the perceptions of intersectoral care.

### Next steps

Next steps include assessing the ICRP for reliability and validity. Once the ICRP Survey is validated, it may be used in evaluation of integrated care to provide feedback to health care providers, as well as for research purposes to assess effectiveness of interventions towards a better integrated health care system. In addition, by translating the ICRP Survey to other languages and settings, the ICRP Survey may be used to compare patient perceptions of intersectoral care between countries. Finally, the ICRP Survey focusing on medical-dental integration may be seen as a blue-print. The survey can be adapted to assess other multidisciplinary health care settings.

### Strengths and limitations

Strengths of this study outweigh identified limitations. This is the first study to develop a questionnaire to assess the patient perceptions of the integration of general and oral health care. For this purpose, we involved an international expert panel representing different expertise areas including primary care, oral care, integration of care, and patient-reported outcome measures, and assessed the resulting framework by involving an independent panel consisting of patients. The conceptual framework and survey were based on a previously validated framework and survey. The ICRP survey was deemed to have good understandability by the patient panel and was successfully used in the pilot study. Main results were that about 80 percent of participants did want their health care providers to coordinate their primary and dental health care, but there was variability in the assumed knowledge and awareness of medical and dental health care providers.

Our study has some limitations. Firstly, the expert panel consisted of seven experts. A larger expert panel might have better reflected the opinions of the involved stakeholder groups such as general practitioners and dentists. However, a panel of seven experts has previously been suggested as being sufficient [28]. Secondly, the expert panel did not involve patient representatives. However, patients were subsequently involved in assessing the understandability and pilot-testing the feasibility of the instrument . Eighteen patients were involved in assessing the survey’s understandability which represents a relevant aspect of content validity. In addition, we did not assess the level of health literacy or level of education of these participants. It is uncertain whether all levels of society were represented in this patient panel. This may have influenced the test for understandability. Thereafter, 199 patients participated in a pilot study which demonstrated the instrument’s feasibility for being administered to the intended target group. Nevertheless, future research should elaborate further on the validity and reliability of the ICRP instrument. In line with the COSMIN Taxonomy of Measurement Properties, the choice of suitable metrics should satisfy validity, reliability, responsiveness, and interpretability [29].

Thirdly, for development of the survey we chose to rely more on group discussion and included discussion rounds before and after the online Delphi assessment round. This decision was made because we believed that this would benefit the aim of the study and ensure a short and simple questionnaire. Due to different time zones, it was challenging to have group discussions with all experts involved. Therefore, some experts could not be involved in all group discussions. However, they provided individual responses after the discussion rounds. Consequently, they were aware of what was previously discussed, but did not have the opportunity to participate in the discussion and the other experts were not able to respond to their comments during the same round. Possibly, this has led to the large number of questions and different formats of the questions included in the online Delphi stage. However, the views of all experts, regardless of their participation in the group discussion or in individual conversations, were included and the final ICRP Survey was sent electronically to all experts to ensure that everyone agreed on the survey.

Fourthly, the patients involved in phase 2 of this study might not have been a reliable representation of the general population. Participants were predominately young and higher educated individuals. In addition, the response rate was unclear. It is uncertain how many patients in the practices were asked to complete the survey but refused, and how many people in the social networks of the researchers saw the invitation to participate. Further assessment of the survey based on a more representative sample of users may therefore be needed.

## Conclusions

The prototypic ICRP Survey provides a short and easy-to-use survey to assess patient perceptions on the integration of oral and primary medical care. It can serve to leverage patient feedback to raise awareness for gaps in the alignment of intersectoral care process and to enhance the integration of primary and oral healthcare. Future research is encouraged to further evaluate the ICRP Survey’s applicability in primary and oral healthcare.

## Additional Files

The additional files for this article can be found as follows:

10.5334/ijic.8933.s1 Appendix I. Questions in round 2 (online round).

10.5334/ijic.8933.s2 Appendix II. Delphi round 2: Ranked questions based on number of times chosen, median and mean (ranked high to low).

10.5334/ijic.8933.s3 Appendix III. Integrated Care Reported by Patients (ICRP) Survey.

## Abbreviations

GP: general practitioner

PPIC survey: Patient Perceptions of Integrated Care survey

ICRP Survey: Intersectoral Care Reported by Patients Survey

PSPIC survey: Provider and Staff perceptions of Integrated Care survey

MHCC survey: Medical Home Care Coordination

## References

[1] Kassebaum NJ, Smith AGC, Bernabe E, et al. Global, Regional, and National Prevalence, Incidence, and Disability-Adjusted Life Years for Oral Conditions for 195 Countries, 1990–2015: A Systematic Analysis for the Global Burden of Diseases, Injuries, and Risk Factors. J Dent Res. 2017;96(4):380–387. DOI: 10.1177/0022034517693566 28792274 PMC5912207

[2] FDI World Dental Federation. *The Challenge of Oral Disease – A call for global action*. The Oral Health Atlas. 2nd ed. Geneva; 2015.

[3] Paulson DR, Ingleshwar A, Theis-Mahon N, Lin L, John MT. The Correlation Between Oral And General Health-Related Quality Of Life In Adults: A Systematic Review And Meta-Analysis. J Evid Based Dent Pract. 2025 Mar;25(1S):102078. DOI: 10.1016/j.jebdp.2024.102078 40087015 PMC11909413

[4] Tan H, Peres KG, Peres MA. Retention of Teeth and Oral Health-Related Quality of Life. J Dent Res. 2016;95(12):1350–1357. DOI: 10.1177/0022034516657992 27466396

[5] Haag DG, Peres KG, Balasubramanian M, Brennan DS. Oral Conditions and Health-Related Quality of Life: A Systematic Review. J Dent Res. 2017;96(8):864–874. DOI: 10.1177/0022034517709737 28581891

[6] Seitz MW, Listl S, Bartols A, Schubert I, Blaschke K, Haux C, Van Der Zande MM. Current Knowledge on Correlations Between Highly Prevalent Dental Conditions and Chronic Diseases: An Umbrella Review. Prev Chronic Dis. 2019 Sep 26;16:E132. DOI: 10.5888/pcd16.180641 31560644 PMC6795069

[7] Botelho J, Mascarenhas P, Viana J, Proença L, Orlandi M, Leira Y, Chambrone L, Mendes JJ, Machado V. An umbrella review of the evidence linking oral health and systemic noncommunicable diseases. Nat Commun. 2022 Dec 9;13(1):7614. DOI: 10.1038/s41467-022-35337-8 36494387 PMC9734115

[8] Herrera D, Sanz M, Shapira L, Brotons C, Chapple I, Frese T, Graziani F, Hobbs FDR, Huck O, Hummers E, Jepsen S, Kravtchenko O, Madianos P, Molina A, Ungan M, Vilaseca J, Windak A, Vinker S. Periodontal diseases and cardiovascular diseases, diabetes, and respiratory diseases: Summary of the consensus report by the European Federation of Periodontology and WONCA Europe. Eur J Gen Pract. 2024 Dec;30(1):2320120. DOI: 10.1080/13814788.2024.2320120 38511739 PMC10962307

[9] Preshaw PM, Alba AL, Herrera D, et al. Periodontitis and diabetes: a two-way relationship. Diabetologia. 2012;55(1):21–31. DOI: 10.1007/s00125-011-2342-y 22057194 PMC3228943

[10] Holmstrup P, Damgaard C, Olsen I, et al. Comorbidity of periodontal disease: two sides of the same coin? An introduction for the clinician. J Oral Microbiol. 2017;9(1):1332710. DOI: 10.1080/20002297.2017.1332710 28748036 PMC5508374

[11] Sheiham A, Watt RG. The common risk factor approach: a rational basis for promoting oral health. Community Dent Oral Epidemiol. 2000;28(6):399–406. DOI: 10.1034/j.1600-0528.2000.028006399.x 11106011

[12] Scully C. Oral adverse effects of drugs. In: Scully C, editor. Scully’s Medical Problems in Dentistry. Vol 7th. Edinburgh: Churchill Livingstone/Elsevier; 2014. pp. 697–698.

[13] Maxey HL, Norwood CW, Weaver DL. Primary Care Physician Roles in Health Centers with Oral Health Care Units. Journal of the American Board of Family Medicine. 2017;30(4):491–504. DOI: 10.3122/jabfm.2017.04.170106 28720630

[14] Lamster IB, Myers-Wright N. Oral Health Care in the Future: Expansion of the Scope of Dental Practice to Improve Health. J Dent Educ. 2017;81(9):eS83–eS90. DOI: 10.21815/JDE.017.038 28864808

[15] Fried JL, Maxey HL, Battani K, Gurenlian JR, Byrd TO, Brunick A. Preparing the Future Dental Hygiene Workforce: Knowledge, Skills, and Reform. J Dent Educ. 2017;81(9):eS45–eS52. DOI: 10.21815/JDE.017.032 28864803

[16] Sippli K, Rieger MA, Huettig F. GPs’ and dentists’ experiences and expectations of interprofessional collaboration: findings from a qualitative study in Germany. BMC Health Serv Res. 2017;17(1):179. DOI: 10.1186/s12913-017-2116-4 28270205 PMC5341464

[17] Singer SJ, Burgers J, Friedberg M, Rosenthal MB, Leape L, Schneider E. Defining and measuring integrated patient care: promoting the next frontier in health care delivery. Medical care research and review. 2011;68(1):112–127. DOI: 10.1177/1077558710371485 20555018

[18] Suter E, Oelke ND, Adair CE, Armitage GD. Ten Key Principles for Successful Health Systems Integration. Healthcare quarterly. 2009;13(Spec No):16–23. DOI: 10.12927/hcq.2009.21092 20057244 PMC3004930

[19] Stewart M, Brown JB, Donner A, et al. The impact of patient-centered care on outcomes. J Fam Pract. 2000;49(9):796–804. 11032203

[20] Thomas J, 3rd, Iyer NN, Collins WB. Associations between perceived chronic care quality, perceived patient centeredness, and illness representations among persons with diabetes. J Healthc Qual. 2014;36(5):50–59. DOI: 10.1111/jhq.12077 25263955

[21] Singer SJ, Friedberg MW, Kiang MV, Dunn T, Kuhn DM. Development and preliminary validation of the Patient Perceptions of Integrated Care survey. Medical care research and review. 2013;70(2):143–164. DOI: 10.1177/1077558712465654 23161612

[22] Zlateva I, Anderson D, Coman E, Khatri K, Tian T, Fifield J. Development and validation of the Medical Home Care Coordination Survey for assessing care coordination in the primary care setting from the patient and provider perspectives. BMC Health Serv Res. 2015;15:226. DOI: 10.1186/s12913-015-0893-1 26113153 PMC4482098

[23] Walker KO, Stewart AL, Grumbach K. Development of a survey instrument to measure patient experience of integrated care. BMC Health Serv Res. 2016;16:193. DOI: 10.1186/s12913-016-1437-z 27250117 PMC4890282

[24] Derrett S, Gunter KE, Samaranayaka A, et al. Development and Testing of the Provider and Staff Perceptions of Integrated Care (PSPIC) Survey. Medical care research and review. 2017:1077558717745936. DOI: 10.5334/ijic.3236 PMC6545265 29231130

[25] Fryer AK, Friedberg MW, Thompson RW, Singer SJ. Patient perceptions of integrated care and their relationship to utilization of emergency, inpatient and outpatient services. Healthc (Amst). 2017.5(4):183–193. DOI: 10.1016/j.hjdsi.2016.12.005 28117243

[26] Dalkey N, Helmer O. An Experimental Application of the Delphi Method to the use of experts. Management Science. 1963;9(3):458–467. DOI: 10.1287/mnsc.9.3.458

[27] Ware JE, Jr., Sherbourne CD. The MOS 36-item short-form health survey (SF-36). I. Conceptual framework and item selection. Med Care. 1992;30(6):473–483. DOI: 10.1097/00005650-199206000-00002 1593914

[28] Fitch K, Bernstein S, Aguilar M, et al. The RAND/UCLA Appropriateness Method User’s Manual. Santa Monica, CA, USA: RAND; 2001.

[29] Mokkink LB, Terwee CB, Patrick DL, Alonso J, Stratford PW, Knol DL, Bouter LM, de Vet HC. The COSMIN study reached international consensus on taxonomy, terminology, and definitions of measurement properties for health-related patient-reported outcomes. J Clin Epidemiol. 2010;63(7):737–745. DOI: 10.1016/j.jclinepi.2010.02.006 20494804

