# Supplementary material for: Development of the Intersectoral Care Reported by Patients Survey for Primary and Oral Healthcare
Source: Int J Integr Care. 2025 Jul 23;25(3):13. doi: 10.5334/ijic.8933 (PMC12292054; doi:10.5334/ijic.8933)
Supplement: Appendix I. — Questions in round 2 (online round). [file ijic-25-3-8933-s1.pdf]

Appendix I. Questions in round 2 (online round)

1. **Are you aware of possible relationships between your dental and general health?**
   - o Yes
   - o No

2. **Do you expect your health care providers to exchange information about your health?**
   - o Yes
   - o No

3. **Do you want your health care providers to exchange necessary information about your health?**
(Necessary information: medical information on your status, treatments, medication use or care gaps)
   - o Yes
   - o No

4. **Do you want your health care providers to coordinate their care based on both your general and dental health?**
   - o Yes
   - o No

5. **A) Do you think your dentist or dental health care provider has enough knowledge about general health?**
(Dental health care provider: anyone who is responsible for monitoring your dental health status and treatment of your dental problems)
   - o Yes
   - o No

5. **B) In your view, how much knowledge does your dentist or dental health care provider have about general health care?**
(Dental health care provider: anyone who is responsible for monitoring your dental health status and treatment of your dental problems)

1	2	3	4	5
No knowledge at all				A lot of knowledge

6. **A) Do you think your dentist or dental health care provider has enough skills for addressing possible relationships between your dental health and your general health?**
(Dental health care provider: anyone who is responsible for monitoring your dental health status and treatment of your dental problems)
   - o Yes
   - o No

6. **B) In your view, how skilled is your dentist or dental health care provider in addressing possible relationships between your dental health and your general health?**
(Dental health care provider: anyone who is responsible for monitoring your dental health status and treatment of your dental problems)

1	2	3	4	5
No skills at all				A lot of skills

7. **In your view, how willing is your dentist or dental health care provider to address possible relationships between your dental health and your general health?**

1	2	3	4	5
Not willing at all				Very willing

8. **A) When did you last visit a dentist or dental health care provider?**

(Dental health care provider: anyone who is responsible for monitoring your dental health status and treatment of your teeth)

- Less than 6 months
- 6-12 months
- 1-2 years
- More than 2 years
- Never visited a dentist or dental health provider

8. B) Did you visit a dentist or dental health care provider in the last 12 months?

(Dental health care provider: anyone who is responsible for monitoring your dental health status and treatment of your teeth)

- Yes
- No

8. C) How often did you visit a dentist or dental health care provider in the last 12 months?

(Dental health care provider: anyone who is responsible for monitoring your dental health status and treatment of your teeth)

- Never
- Once
- Twice
- More than two times

9. A) Do you think your dentist or dental health care provider is up-to-date regarding your general health?

(Dental health care provider: anyone who is responsible for monitoring your dental health status and treatment of your dental problems

- Yes
- No

9. B) How confident are you that your dentist or dental health care provider is aware of your medical history?

(Dental health care provider: anyone who is responsible for monitoring your dental health status and treatment of your teeth)

1	2	3	4	5
Not confident at all				Very confident

10. A) Did your dentist or dental health care provider ever discuss your general health with you?

(Dental health care provider: anyone who is responsible for monitoring your dental health status and treatment of your teeth)

- Yes
- No

10. B) How often has your dentist or dental health care provider discussed your general health with you?

(Dental health care provider: anyone who is responsible for monitoring your dental health status and treatment of your teeth)

- Never
- Sometimes
- Usually
- Always

11. A) During your most recent dental visit, did your dentist or dental health care provider ask about changes in your general health?

(Dental health care provider: anyone who is responsible for monitoring your dental health status and treatment of your teeth?)

(Changes in general health: questions related to this could for example be about your medical history, visits to the general practice or the hospital, reasons for visiting an health care provider and whether certain medical tests were performed)

- Yes
- No

11. B) During your most recent dental visit, did your dentist or dental health care provider ask about one of the following aspects: your medical history, visit to the general practice, visit to the hospital, reasons for visiting an health care provider, any medical test results, changes in your medication?

(Dental health care provider: anyone who is responsible for monitoring your dental health status and treatment of your teeth)

- Yes
- No

12. How would you rate your general health?

Possible answer categories:

1	2	3	4	5
Very poor				Very good

Or

- Very unhealthy
- Unhealthy
- Healthy
- Very healthy

Or

- Very poor
- Poor
- Moderate
- Good
- Very good
- Excellent

13. Do you use any medication?

- Yes
- No

14. Who is your primary health care provider?

(Primary health care provider: this is the health care provider that you (the patient) see as being the most responsible for you general health care)

- General practitioner
- Specialist, namely …

15. A) Do you think your general practitioner or primary health care provider has enough knowledge about dental health in general?

- Yes
- No

15. B) In your view, how much knowledge does your general practitioner or primary health care provider have about dental health care?

1	2	3	4	5
No knowledge at all				A lot of knowledge

16. A) Do you think your general practitioner or primary health care provider has enough skills for addressing possible relationships between your dental health and your general health?

- Yes
- No

16. B) In your view, how skilled is your general practitioner or primary health care provider in addressing possible relationships between your dental health and your general health?

1	2	3	4	5
No skills at all				A lot of skills

17. **In your view, how willing is your general practitioner or primary health care provider to address possible relationships between your dental health and your general health?**

1	2	3	4	5
Not willing at all				Very willing

18. **A) When did you last visit your general practitioner or primary health care provider?**
    - Less than 6 months
    - 6-12 months
    - 1-2 years
    - More than 2 years
    - Never visited a general practitioner or primary health care provider

18. **B) Did you visit a general practitioner or primary health care provider in the last 12 months?**
    - Yes
    - No

18. **C) How often did you visit a general practitioner or primary health care provider in the last 12 months?**
    - Never
    - Once
    - Twice
    - More than two times

19. **A) Do you think your general practitioner or primary health care provider is up-to-date regarding your dental health?**
    - Yes
    - No

19. **B) How confident are you that your general practitioner or primary health care provider is aware of your dental history?**

1	2	3	4	5
Not confident at all				Very confident

20. **A) Did your general practitioner or primary health care provider ever discuss your dental health with you?**
    - Yes
    - No

20. **B) How often has your general practitioner or primary health care provider discussed your dental health with you?**
    - Never
    - Sometimes
    - Usually
    - Always

21. **A) During the past year, did your general practitioner or primary health care provider ask about changes in your dental health?**

(Changes in dental health: questions related to this could for example be about your dental history, visits to the dentist or dental clinic, reasons for visiting a dental health care provider and whether certain dental tests were performed)
- Yes
- No

21. **B) During the past year, did your general practitioner or primary health care provider ask about one of the following aspects: your dental history, visits to the dentist or dental clinic, reasons for visiting a dental health care provider, results from dental tests, dental treatments?**
    - Yes
    - No

22. How would you rate your dental health?
Possible answer categories:

1	2	3	4	5
Very poor				Very good

Or
- Very unhealthy
- Unhealthy
- Healthy
- Very healthy

Or
- Very poor
- Poor
- Moderate
- Good
- Very good
- Excellent

23. A) How confident are your filling out medical forms by yourself?

1	2	3	4	5
Not confident at all				Very confident

23. B) For me to discuss all my health care needs with all my healthcare providers is:
Slider:
0 ———————————————— 100
Very difficult — Very easy

23. C) I am certain that I can discuss all my health care needs with my health care providers
Possible answer categories:
Slider:
0 ———————————————— 100
Completely disagree — Completely agree

Or

1	2	3	4	5
Completely disagree				Completely agree

24. A) The dentist has nothing to do with general health

1	2	3	4	5
Completely disagree				Completely agree

24. B) The dentist does not know much about general health

1	2	3	4	5
Completely disagree				Completely agree

24. C) The general practitioner has nothing to do with dental health

1	2	3	4	5
Completely disagree				Completely agree

24. D) The general practitioner does not know much about dental health

1	2	3	4	5
Completely disagree				Completely agree

25. Which type(s) of health information do you want your health care providers to communicate about?
Multiple answers possible
- All information
- Diagnosis
- Results of medical tests
- Medication
- Medical history
