# Supplementary material for: Development of the Intersectoral Care Reported by Patients Survey for Primary and Oral Healthcare
Source: Int J Integr Care. 2025 Jul 23;25(3):13. doi: 10.5334/ijic.8933 (PMC12292054; doi:10.5334/ijic.8933)
Supplement: Appendix II. — Delphi round 2: Ranked questions based on number of times chosen, median and mean (ranked high to low). [file ijic-25-3-8933-s2.pdf]

Appendix II. Delphi round 2: Ranked questions based on number of times chosen, median and mean (ranked high to low)

**Questions and answer options**	**# Chosen**	**Median**	**Mean**
Do you want your health care providers to coordinate their care based on both your general and dental health?<br>*(Yes/No)*	5	8	7.857
Which type of health information do you want your health care providers to communicate about?<br>*(All information; relevant diagnosis; results of medical tests; medication; infectious diseases; medical history)*	4	8.5	8.5
Are you aware of possible relationships between your dental and general health?<br>*(Yes/No)*	3	9	8.143
How would you rate your general health?<br>*(Very poor; poor; moderate; good; very good; excellent)*	3	8.5	8.167
During your most recent dental visit, did your dentist or dental health care provider ask about changes in your general health?<br>*(Yes/No)*	3	8	7.833
During your most recent dental visit, did your dentist or dental health care provider ask about one of the following aspects: your medical history; visit to the general practice; visit to the hospital; reasons for visiting a health care provider; any medical test results; changes in your medication?<br>*(Yes/No)*	3	7.5	7.167
When did you last visit a dentist or dental health care provider?<br>*(Less than 6 months; 6-12 months; 1-2 years; more than 2 years; never visited a dentist or dental health care provider)*	3	7	7.143
During the past year, did your general practitioner or primary health care provider ask about changes in your dental health?<br>*(Yes/No)*	2	9	8.667
During the past year, did your general practitioner or primary health care provider ask about one of the following aspects: your dental history; visits to the dentist or dental clinic; reasons for visiting a dental health care provider; results from dental tests; treatment of gums; surgery/extractions in the mouth; renewal of prostheses; crowns or fillings; root treatments?<br>*(Yes/No)*	2	9	8.167
How confident are you that your general practitioner or primary health care provider is aware of your dental history?<br>*(Likert scale from "Not confident at all" to "Very confident" or "Fully disagree" to "Fully agree")*	2	8.5	8.333
In your view, how much knowledge does your general practitioner or primary health care provider have about dental health care?<br>*(Likert scale from "No knowledge at all" to "A lot of knowledge")*	2	8.5	8.167
In your view, how willing is your general practitioner or primary health care provider to address possible relationships between your dental health and your general health?<br>*(Likert scale from "Not willing at all" to "Very willing")*	2	8	8
In your view, how skilled is your dentist or dental health care provider in addressing possible relationships between your dental health and your general health?<br>*(Likert scale from "No skills at all" to "A lot of skills")*	2	8	7.714

Do you think your dentist or dental health care provider is up-to-date regarding your general health? *(Yes/No)*	2	8	7.429
I am certain that I can discuss all my health care needs with my health care providers *(Likert scale from "Fully disagree" to "Fully agree")*	2	7.5	7.333
Do you think your dentist or dental health care provider has enough skills for addressing possible relationships between your dental health and your general health? *(Yes/No)*	2	7	5.571
Did your general practitioner or primary health care provider ever discuss your dental health with you? *(Yes/No)*	1	9	8.667
How often has your general practitioner or primary health care provider discussed your dental health with you? *(Never; sometimes; usually; always)*	1	9	8.5
How would you rate your dental health? *(Very poor; poor; moderate; good; very good; excellent)*	1	9	8.5
Do you expect your health care providers to exchange information about your health? *(Yes/No)*	1	9	7.714
How confident are you that your dentist or dental health care provider is aware of your medical history? *(Likert scale from "Not confident at all" to "Very confident")*	1	9	7.143
When did you last visit your general practitioner or primary health care provider? *(Less than 6 months; 6-12 months; 1-2 years; more than 2 years; never visited a general practitioner or primary health care provider)*	1	8	8.333
How often has your dentist or dental health care provider discussed your general health with you? *(Never; sometimes; usually; always)*	1	8	8
How often did you visit a general practitioner or primary health care provider in the last 12 months? *(Never; once; twice; more than two times)*	1	8	7.833
The dentist has nothing to do with general health *(Likert scale from 'completely disagree' to 'completely agree')*	1	8	7.833
Do you want your health care providers to exchange necessary information about your health? *(Yes/No)*	1	8	7.714
Do you think your general practitioner or primary health care provider is up-to-date regarding your dental health? *(Yes/No)*	1	8	7.667
In your view, how skilled is your general practitioner or primary health care provider in addressing possible relationships between your dental health and your general health? *(Likert scale from 'No skills at all' to 'A lot of skills')*	1	8	7.667
How confident are you filling out medical forms by yourself? *(Likert scale from 'Not confident at all' to 'Very confident')*	1	7.5	6.333
The general practitioner has nothing to do with dental health *(Likert scale from 'completely disagree' to 'completely agree')*	1	7	7.167

Do you use any medication?<br>*(Yes/No)*	1	7	7
For me to discuss all my health care needs with all my health care providers is:<br>*(Slider from 'very difficult' (0) to 'very easy' (100))*	1	7	6.333
In your view, how willing is your dentist or dental health care provider to address possible relationships between your dental health and your general health?<br>*(Likert scale from 'Not willing at all' to 'Very willing')*	1	6	5.286
How often did you visit a dentist or dental health care provider in the last 12 months?<br>*(Never; once; twice; more than two times)*	1	5	4.714
In your view, how much knowledge does your dentist or dental health care provider have about general health care?<br>*(Likert scale from 'No knowledge at all' to 'A lot of knowledge')*	0	9	8
Do you think your general practitioner or primary health care provider has enough knowledge about dental health in general?<br>*(Yes/No)*	0	8	8
The dentist does not know much about general health<br>*(Likert scale from 'completely disagree' to 'completely agree')*	0	7.5	7.333
The general practitioner does not know much about dental health<br>*(Likert scale from 'completely disagree' to 'completely agree')*	0	7.5	7.167
Do you think your general practitioner or primary health care provider has enough skills for addressing possible relationships between your dental health and your general health?<br>*(Yes/No)*	0	7.5	6.333
Did your dentist or dental health care provider ever discuss your general health with you?<br>*(Yes/No)*	0	7	6.429
Do you think your dentist or dental health care provider has enough knowledge about general health?<br>*(Yes/No)*	0	7	5.571
Did you visit a general practitioner or primary health care provider in the last 12 months?<br>*(Yes/No)*	0	6.5	6.333
Who is your primary health care provider?<br>*(General practitioner; Specialist; namely …)*	0	6	5.333
Did you visit a dentist or dental health care provider in the last 12 months?<br>*(Yes/No)*	0	5	4.857
