# Supplementary material for: Development of the Intersectoral Care Reported by Patients Survey for Primary and Oral Healthcare
Source: Int J Integr Care. 2025 Jul 23;25(3):13. doi: 10.5334/ijic.8933 (PMC12292054; doi:10.5334/ijic.8933)
Supplement: Appendix III. — Integrated Care Reported by Patients (ICRP) Survey. [file ijic-25-3-8933-s3.pdf]

Appendix III. Integrated Care Reported by Patients (ICRP) Survey

This short survey of 13 questions assesses the perceptions of patients about the integration of health care they receive. It gives a general idea of how much the participants want their health care to be integrated as well as how they perceive the integration of health care they receive.
This survey can be used as a stand-alone survey or as an additive to other questionnaires. Completing the survey should take no more than a few minutes.

Definitions used in the ICRP survey:

- Integration of health care: collaboration in health care between different healthcare professionals around the same patient. With well-integrated care, the information about the patient is accessible to every care provider within the team. For example: the general practitioner can vies the dentist's file and vice versa. Because every healthcare provider is aware of the patient's medical record, they can offer their own expertise to the patient in the most effective way.
- Oral health: the ability to speak, smile, taste, touch, chew, swallow and convey a range of emotions through facial expressions with confidence and without pain, discomfort and disease of the mouth, teeth, gums, throat, jaw or face.
- General health: a state of complete physical, mental and social well-being. Note: this is more than just the absence of disease or infirmity.
- General practitioner or primary healthcare provider: a doctor or other healthcare provider involved in the care of your general health. They can act as the first doctor or other provider you see who may then refer you to other medical services.
- Healthcare providers: institutions, doctors, nurses, and all other individuals who provide medical care.
- Dentist or oral healthcare provider: all healthcare providers involved mainly in the care of your teeth, gums and mouth.
- Medical history: the whole series of past illnesses and treatments you have had.
- Social history: all data related to social life that could be important for the treatment, such as major events un the personal environment (divorce, death of a family member, dismissal, etc.)
- Dental history: the whole series of past illnesses and treatments in the mouth, teeth or gums you have had.

1. How aware are you of possible relationships between your oral and general health?

1	2	3	4	5
Not at all aware	Slightly aware	Somewhat aware	Moderately aware	Extremely aware

2. Do you want your healthcare providers to coordinate your care based on both your general and oral health? (And for example exchange among themselves all information they consider necessary)

- o Yes
- o No
- o I do not know

3. Which type of health information do you want your healthcare providers to communicate about?
Multiple answers are possible:

- □ Recent diagnosis
- □ Results of medical tests
- □ Medication
- □ Infectious diseases
- □ Medical history
- □ Social history
- □ Emotional state, for example, sadness
- □ Mental health
- □ I do not want my healthcare providers to communicate
- □ I do not know
- □ Other, namely …

4. In your view, how much knowledge should your dentist or oral healthcare provider have about general health care? (Such as being aware of symptoms and disease in the body, outside the mouth)

1	2	3	4	5
Not knowledgeable at all	Slightly knowledgeable	Moderately knowledgeable	Very knowledgeable	Extremely knowledgeable

5. When did you last visit a dentist or oral healthcare provider?
One answer is possible:
- o Less than 6 months ago
- o 6-12 months ago
- o 1-2 years ago
- o 2-10 years ago
- o Never visited a dentist or dental healthcare provider in the last 10 years

6. To what extent do you agree with the following statement: My dentist or oral healthcare provider is aware of my medical history. (He/she knows for example which diseases I have and takes this into account)

1	2	3	4	5
Strongly disagree	Disagree	Neither agree nor disagree (I do not know)	Agree	Strongly agree

7. During your most recent dental visit, did your dentist or oral healthcare provider ask about one of the following aspects:
Multiple answers are possible:
- □ Your medical history
- □ Your social history
- □ Visit to the general practice
- □ Visit to the hospital
- □ Reasons for visiting a healthcare provider (general practitioner or hospital)
- □ Medical test results
- □ Changes in your medication (including prescriptions as well over the counter medication)
- □ Changes in your general health
- □ None
- □ I do not know or do not remember
- □ Other, namely: …

8. How would you rate your general health?
One answer is possible:
- o Poor
- o Fair
- o Good
- o Very good
- o Excellent

9. In your view, how much knowledge should your general practitioner or primary healthcare provider have about oral health care? (Such as being aware of symptoms and disease inside the mouth)

1	2	3	4	5
Not knowledgeable at all	Slightly knowledgeable	Moderately knowledgeable	Very knowledgeable	Extremely knowledgeable

10. When did you last visit your general practitioner or primary healthcare provider?
One answer is possible:

- ○ Less than 6 months ago
- ○ 6-12 months ago
- ○ 1-2 years ago
- ○ 2-10 years ago
- ○ Never visited a general practitioner or primary healthcare provider in the last 10 years

11. To what extent do you agree with the following statement: My general practitioner or primary healthcare provider is aware of my dental history (He/she knows for example which problems I have in my mouth and takes this into account)
5-point Likert scale:

1	2	3	4	5
Strongly disagree	Disagree	Neither agree nor disagree	Agree	Strongly agree

12. During the past year, did your general practitioner or primary healthcare provider ask about one of the following aspects? (Regardless of which complaints you had)
Multiple answers are possible:
- □ Your dental history
- □ Visits to the dentist or dental clinic
- □ Reasons for visiting a dentist or dental healthcare provider
- □ Dental screening/exam
- □ Dental treatment (e.g. treatment of gums; plaque removal; surgery/extractions in the mouth; renewal of prostheses; crowns, fillings or root canal treatments)
- □ Problems with your mouth, teeth, gums or jaw. (For example pain in the mouth, gums or jaw; problems with chewing, eating, speaking or bleeding gums)
- □ None
- □ I do not know or do not remember
- □ Other, namely …

13. How would you rate your oral health?
One answer is possible:
- ○ Poor
- ○ Fair
- ○ Good
- ○ Very good
- ○ Excellent
